# On the Rudimentary Reproduction of Extremities after Their Spontaneous Amputation

**Published:** 1856-01

**Authors:** 

**Affiliations:** Edinburgh


*On the Rudimentary Reproduction of Extremities after their Spontaneous Amputation.* By Prof. SIMPSON, of Edinburgh.

On the stumps of limbs that have seemingly undergone an early spontaneous amputation in utero, there is often seen a species of anormal structure, which has not as yet, so far as I am aware, been described in any existing work on the subject of monstrosities I allude to the appearance on the ends of many such stumps of a projecting mass, or nodule, varying in size from a small cutaneous ridge to the bulk of a walnut, and having protruding from its surface one, two or more still smaller fleshy divisions of projections, which are provided at their extreme points with nails.

This variety of anormal structure is by no means rare. Several years ago, while searching for instances of it, I found five or six living examples in Edinburgh and its neighborhood; and I have seen some, and heard of many more, living in different parts of Scotland and England. It is interesting, however, not so much for the frequency with which it is met with, as from the nature of the anormal structure itself, consisting, as I believe it does, of a tendency in the human subject to the reproduction of a lost extremity.

As a general law, the power of repairing and reproducing lost parts decrease as we ascend from the lower to the higher parts of the animal scale. In the lowest and simplest forms of animal life, as in polypes, not only are separate parts or segments rapidly restored, but the separate segments themselves sometimes become developed into whole and perfect individuals. A hydra was cut at different times into various portions by Trembley, and fifty separate individuals of the species were developed from the segments of one. Johnson and Duges have shown that animals with a much higher organization, viz: the planariæ—could in the same way be multiplied by artificial subdivision; and Lyonnet and Bonnet found the same true of the nails. As we ascend upward in the scale of life, all power of self-development in separated parts of segments disappears, but the power of regenerating these lost parts or segments is retained to a greater or less degree by the general body of the animal. When the arms or rays of a star-fish are broken off artificially, or when they are thrown off, as they sometimes are in the lingthorn, or Iludia, etc., by a true "spontaneous amputation" on the part of the animal, the lost arms are betimes entirely restored. In crustacea a separated or amputated limb is also rapidly renovated. The head or anterior rings of the earthworm and other annelida are generally regenerated after their decapitation; and the power of reproduction is still so great in the mollusca, that the snail, according to Schweigger, has sometimes its head and antennæ restored after they are removed by amputation, provided the cephalic ganglion lying above œsophagus be left uninjured. In the lower divisions of the vertebrata we have the salamander still capable of re-producing an entire leg or tail, or even of forming a new under jaw; and the triton can regenerate, as in Blumenbach's experiments, a complete and perfect eye. But in the higher and warm-blooded vertebrata this power of repairing and restoring lost compound parts and organs seems totally, or almost totally, wanting. In man, not only are complex individual parts, however small, generally held incapable of restoration, but portions of the higher individual tissues, even, as mucous membrane, muscle, &c., when cut, removed or destroyed, are not usually regenerated in their entire organization. To this general law, however, there are the following exceptions in the human subject:

1st. When the part removed is primitively of a lower type of organization than that of the general body, restoration sometimes occurs. Thus, in a case of a child born with an additional thumb, or with a thumb double from the first joint, the outer or smaller one was amputated by Mr. White, of Manchester. It grew again, and along with it the nail. Subsequently, Mr. Bromfield, of London, a second time carefully removed this superadded portion of thumb, and turned the ball of it fairly out of the socket. "Notwithstanding this," adds Mr. White, "it grew again and a fresh nail was formed."*

2d. In those animals that possess, in the most marked degree, the power of readily regenerating lost compound parts, this power resides especially in the extreme points of the body, as the tail and limbs. In the human subject we sometimes find instances of an appearance of the same power in the extreme parts, as the fingers or toes. I have seen a distinct but imperfect nail grow on the end of the second phalanx of the finger, after the complete amputation of the first phalanx. Similar instances of nails, and consequently of the matrices of these nails, becoming regenerated on the tips of fingers amputated through their first joint, have been recorded by Corvisart, Ansiaux, Blumenbach and others.

3d. When, in the human subject, the removal of a compound part—such as a portion of the extremity—is effected in early fœtal life, and consequently at a time when the physiological powers of the young human being are more assimilated to the reparative and other powers of animals of a lower type in the animal scale, the lost part seems capable of at least a partial and rudimentary restoration. In the animal kingdom generally, we find the power of regeneration greater in the inverse ratio of the degree of development or age of the individual. The more perfect hexapod insects never reproduce a lost limb; but in the larvæ of these same insects, limbs and antennæ are restored after their removal. The experiments of Heineken show that the arachnida, in the same way, lose the property of regenerating their legs after they have ceased to change their skin, and have reached their full or adult development. It is only in the young frog that reproduction of a limb occurs; and Spallanzani found that the rapidity with which the tail of the tadpole and the limbs of the sala-

*Regeneration of Animal and Vegetable Substances, p. 16.

mander are regenerated, was always in an inverse ratio to the age of the animal. So while in the human subject after birth we never see any trace of the reproduction of a limb after amputation, we have the contrary, as I believe, evidence of the possibility of their rudimentary regeneration in the appearances sometimes seen on the ends of stumps resulting from spontaneous amputation in early fœtal or embryonic life.

In most of the cases in which I have observed this appearance of a rudimentary regeneration of an extremity, the spontaneous amputation had occurred in the upper half of the forearm; and the general resemblance of these cases to each other is very remarkable. Usually the rounded end of the limb has exactly the appearance of a stump after amputation, and is well covered with soft parts. Two points of the skin, or rather of the subcutaneous tissue, are found adherent to the ends of the ulna and radius, and present a depressed or umbilicated form, particularly when the forearm is flexed or moved, and the fissures of the skin seen in converging lines to these two points as centres. Midway, and a little in front of these two points, the rudiment of the regenerated extremity is situated in the form of a raised cutaneous fold or fleshy mass or tubercle, and having on its surface one, two or more smaller projections or nodules, furnished with minute nails. In the instance of a young woman of 18 years of age, four such imperfect fingers were seen, two of them tipped with nails. In this, as in most other cases, the left arm is the seat of the mutilation, but I have seen the right similarly affected.

The stump of the left forearm of a fœtus of the seventh month, is preserved in the Obstetric Museum of the University of Edingburgh, having five small rudimentary fingers tipped with minute nails in the usual position on the end of the stump. But the case is principally remarkable for the circumstance, that the cicatrization over the ends of the ulna and radius is not complete. There is an aperture at the end of the radius, through which the end of the bone can be felt when the point of a pin is passed through it. The ulna projects to the cutaneous surface of the stump, and has a small wound or circle of uncovered granulations still around it; or in other words, the cicatrix of the stump is as yet incomplete.—*N. O. Medical News & Hospital Gazette.*

